# Vector competence of the tick *Ixodes sinensis* (Acari: Ixodidae) for *Rickettsia monacensis*

**DOI:** 10.1186/s13071-014-0512-8

**Published:** 2014-11-19

**Authors:** Xiaodong Ye, Yi Sun, Wendong Ju, Xin Wang, Wuchun Cao, Mingyu Wu

**Affiliations:** State Key Laboratory of Pathogen and Biosecurity, Beijing Institute of Microbiology & Epidemiology, No. 20 Dongdajie Str. Fengtai District, Beijing, People Republic of China; Centre for Disease Control and Prevention of Jindong, No.295 Jiangjun Road, Jinhua, Zhejiang province People Republic of China; Center for Health Inspection, Heilongjiang Bureau of Entry & Exit Inspection and Quarantine, No.9 Ganshui Road, Harbin, People Republic of China; Center for Disease Control and Prevention of Wenzhou, No. 59 Yingdaoguan Rd., Lucheng District, Wenzhou, Zhejiang province People Republic of China

**Keywords:** *Rickettsia monacensis*, *Ixodes sinensis*, Vector competence

## Abstract

**Background:**

Cases of Mediterranean Spotted Fever like rickettsioses, caused by *Rickettsia monacensis*, have become more common in the last 10 years. In China, natural infection of *R. monacensis* in various tick species has been confirmed but the vector(s) of *R. monacensis* have not been recorded.

**Methods:**

The prevalence of *R. monacensis* in >1500 Ixodidae ticks from central and southern China was determined using centrifugation-shell vial culture and polymerase chain reaction techniques. The predominant species, *Ixodes sinensis,* harbored a natural infection of *R. monacensis* and was assumed to be a vector candidate of *R. monacensis*. Experimental transmissions were initialized by infecting *Rickettsia*-free tick colonies with *R. monacensis* using capillary tube feeding (CTF) or immersion techniques. Transstadial and transovarial transmissions, and transmission from ticks to mice, were conducted under laboratory conditions.

**Results:**

*R. monacensis* was isolated and identified from hemolymph of *Ixodes sinensis* using molecular techniques. Transovarial transmission of *R. monacensis* from infected ♀*I. sinensis* to offspring was documented and infected offspring successfully passed *Rickettsia* to mice. Transstadial transmission rates were 58% in larva to nymph and 56% in nymph to adult stages. Infected nymphs and adults were also able to infect mice.

**Conclusions:**

*I. sinensis* is a competence vector for *R. monacensis* as demonstrated by natural infection and transmission studies.

## Background

Rickettsiae are obligate intracellular, gram-negative, alpha-proteobacteria usually transmitted by arthropod vectors.They cause various human diseases including emerging spotted fever rickettsiosis [1]. Since the initial study of the spotted fever group (SFG) *rickettsia* by Ricketts (1906) more than 27 described species and uncharacterized strains have been associated with spotted fever rickettsiosis [2,3]. *R. monacensis* was first isolated and characterized in 2002 from *Ixodes ricinus* ticks collected in Munich, Germany [4]. Five years later, *R. monacensis* was identified from Mediterranean Spotted Fever (MSF)-like patients in Spain [5]. General discomfort, headache, and joint pain, a nonpruritic, disseminated maculopapular rash or an erythematous rash with no inoculation escharare typical symptoms. Since 2007, MSF-like cases have been documented in Italy [6], Croatia [7] and the Republic of Korean (EU883092, FJ009429). To determine possible arthropod vectors, vertebrate reservoirs, and geographic ranges, epidemiological surveys for *R. monacensis* have been performed in several European countries [8-11]. The prevalence of *R. monacensis* in *I. ricinus* ranged from 4% (Spain), 8.6% (Germany), and 12.2% (Slovakia) to 52.9% (Bulgaria) [12,13]. *R. monacensis* was also found in *Ixodes persulcatus* from mainland China [14], *Ixodes nipponensis* [15] and *Haemaphysalis longicornis* [16] from the Republic of Korea, and *Haemaphysalis punctata* [8] from Italy, suggesting that many tick species are involved in the zoonotic cycles and wide geographic range of *R. monacensis*. However, the presence of *R. monacensis* in these ticks does not prove that they are competent vectors of *R. monacensis*. Transmission data provides better evidence for the potential of Ixodid ticks to serve as vectors for *R. monacensis*.

Tick-borne rickettsial diseases are a significant problem in China. Over the last 5 years, tick populations have generally increased and this has led to an increase in human tick bites [17]. Two phenomena are striking. First, thousands of hospitalized patients have unexplained febrile illnesses coinciding with the period of greatest tick activity. Data on clinical symptoms, history of exposure to ticks, and presumptive therapy strongly suggests that some of the patients are infected by SFG rickettsial pathogens [17]. Second, the range of human cases appears to be expanding southward. Many human cases with typical spotted fever symptoms have been found in central and southern China. While some common factors may be at play, the mechanisms behind infection and range expansion have not been fully clarified. Infections may be influenced by climate changes, potential vector ticks, host population dynamics, and human behaviour changes. Due to current diagnostic techniques that depend on clinical symptoms and serological and/or commercially available genus-specific PCR assays, detailed information about the pathogens and the vector ticks is scarce.

Knowledge of tick borne rickettsiosis ecology is essential to understand the potential threat of emerging *Rickettsia* spp. and vector ticks in central and southern China.To address this issue, we surveyed for *Rickettsia* spp. in Chinese Ixodid ticks. Our focus was on species which frequently bite tourists and residents and are prevalent throughout pastoral and forest areas from Henan, Hubei, Anhui, Shandong, Jiangsu and Zhejiang provinces. The survey was conducted during 2009–2013. *R. monacensis* was successfully isolated and identified from *I. sinensis* in Guangshan county, Henan province. *I. sinensis* is closely related to the well-known vector *I. ricinus* inmorphology, phylogeny and blood feeding behavior. *I. sinensis* was therefore assumed to be a probable vector of *R. monacensis* and its capability for transtadial and transovarial transmission was determined.

## Methods

### Ticks

**Figure 1:**
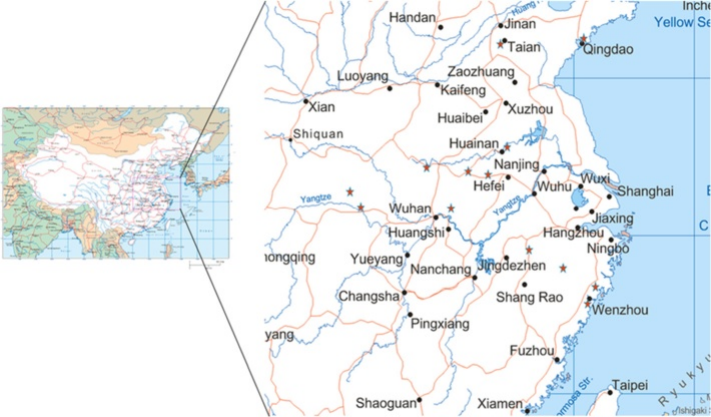
**Origins of ticks in the study.** Ticks collected from January, 2009 to January 2013 in sites marked with stars on the map.

Questing ticks were collected on vegetation using the blanket sweeping method in the survey sites from January, 2009 to January 2013 (Figure 1).After identification using standard taxonomic keys, hemolymph, for culture of *R. monacensis*, was collected from each tick from a cut leg using a sterile capillary tube [18]; the body of each tick was simultaneously screened for presence of the pathogen. To obtain a *Rickettsia* free colony, engorged females *I. sinensis* were maintained individually in 1.5 cm diameter test tubes for egg-laying under a 16 hr light/8 hr dark photoperiod at 22°C within glass desiccators above a saturated aqueous solution of K_2_SO_4_ that maintained 97% RH [19]. After hatching, about 50 filial larvae were sampled from each adult. Both sampled filial larvae and their mother were tested for *Rickettsia* spp. by PCR screening described as follows. Only the *Rickettsia*-negative filial progeny from *Rickettsia* free mothers were used in the experimental colony. The *Rickettsia* free nymph and adult colonies were harvested from *Rickettsia* free larvae and nymph colonies fed on pathogen free C_3_H mice.

### C_3_H mice

Pathogen free, 14-day-old, male C_3_H mice were provided by the Animal Care Laboratory of the Institute of Zoology, Chinese Academy of Science and served as hosts for both *I. sinensis* and *R. monacensis*. C_3_H mice were maintained in accordance with the Institutional Animal Care and Use Committee of Beijing Institute of Microbiology and Epidemiology.

### Cultivation *R. monacensis* from ticks

Hemolymph from ticks was cultured in human embryonic lung (HEL) fibroblasts with the centrifugation-shell vial technique using 12-mm round cover slips seeded with 1 ml of medium containing 50,000 cells and incubated in a 5% CO_2_ incubator at 37°C for 3 days to obtain a confluent monolayer [20]. Cultures were monitored for 4 weeks, and bacterial growth was assessed every 7 days on cover slips directly inside the shell vial using Gimenez and immunofluorescence staining methods. For positive cultures, the *Rickettsia* isolate was identified using PCR and sequencing as described below.

### Infection *I. sinensis* with CTF and immersion methods

*Rickettsa*-free *I. sinensis* were fixed on slides individually using double-sided adhesive tape. A drawn-our capillary tube filled with 10 μL of medium containing 500 cells infected with *R. monacensis* was placed over hypostomes of tick and the tick was allowed to feed at 34°C as described for artificial infection by *Borrelia burgdorferi* [21]. Pipettes were replaced every 2–3 h for 6 h and then ticks were detached from the double-sided tape and returned to colony maintenance conditions. A similar procedure was performed to infect the *Rickettsia* free colony of nymphal *I. sinensis.* To infect larvae by immersion, 50 μL of a medium containing 2,500 cells infected with *R. monacensis* was cracked with an ultrasonic cell disruptor (800 watt, 2 h) prior to the immersion procedures. Ticks were placed in this medium and vortexed at medium speed and incubated at 34°C for 30 min. To avoid larval flotation the centrifugation was pulsed. Centrifuged larvae were surface disinfected by immersion in a 0.1% bleach solution for 2 min, washed in distilled H_2_O, and returned to colony maintenance conditions [22].

### Transovaries Transmission, TOT

After infection by CTF, every 4 *I. sinensis* females were allowed to feed on one naïveC_3_H mouse along with 4 males. To avoid grooming, each mouse was restrained with a collar. The parasitized mice were reared individually in a cage over water pans, where well fed females were recovered and returned to colony maintenance conditions after dropping from their hosts. The engorged females were maintained individually until egg laying. From each maternal individual, we sampled 300 F_1_ eggs and allocated them randomly into 3 pools. The rest of the eggs were kept in colony maintenance conditions to hatch. After hatching, 300 F_1_ larvae were sampled as before and allocated to 3 pools. The filial eggs and resultant larvae pools were submitted to be screened for *R. monacensis* infection with the PCR and sequencing method described below.

### Transstadial Transmission, TS

After infection by CTF or immersion methods, 10 nymphs or 50 larvae of *I. sinensis* were allowed to feed on one C_3_H mouse as described above. A total of 15 C_3_H mice were used as hosts for nymphs and 6 mice were used for larvae. After detachment, the engorged nymphs and larvae were harvested. A total of 30 engorged nymph or 30 engorged larvae were sampled and the rest were maintained individually prior to molting into adult or nymphs. Then, the subsequent 25 males and 25 females or 50 nymphs were also sampled and the rest were used in the following experiment to study transmission from tick to host reservoir. The sampled ticks were tested for *R. monacensis* infection with PCR and sequencing methods as described below.

### Transmission from tick to host reservoir

The transmission competence of tick to naïve mice was tested as follows. Females, nymphs and larvae of *I. sinensis* obtained from TS and TOT experiments were also allowed to feed on naïve C_3_H mice respectively as described above. At 5 days following tick detachment, blood was collected by tail vein from each mouse to evaluate for *R. monacensis* infection using PCR and sequencing methods.

### PCR detection for *Rickettsia monacensis* in cells, tick and mice and sequencing

The QIAamp DNA mini Kits (QIAGEN, Hilden, Germany) were utilized to prepare DNA templates from the cells, ticks and mice samples according to the manufacturer’s protocol. All PCR assays used Taq polymerase (Promega) in 50 μL reactions with the manufacturer’s suggested buffer and nucleotide concentrations. Presence of rickettsial DNA in tick and mice blood extracts was detected with specific primers as follows: Primer GltA.877p and GltA1258n [23], which amplify a 382 bp part of *glt*A gene; Primer Rr17.61 and Rr17.492 [24], which amplify a 438 bp fragmentof17kD protein gene; Primer Rr70p and Rr602nfor the 530 bp fragment of the *omp*A gene; RrompBf and RrompBr for a 515 bp fragment of *omp*A [23]. The PCR were performed as described previously [23] with distilled water instead of DNA template used as a negative control. All amplicons were cloned into the pGEM-T Easy vector and subjected to bidirectional sequencing (Sangon Biotech, Shanghai, China) with SP6 and T7 promoter primers. The newly obtained sequences were aligned with corresponding sequences retrieved from the GenBank database (http://www.ncbi.nlm.nih.gov) using BioEditv.7.0.5.3. The phylogenetic trees for the genes were constructed applying the Neighbour-Joining (NJ) algorithm implemented in the software package MEGA 5.20.

### Ethics statement

The study had received the specific approval of the Institutional Animal Care and Use Committee (IACUC) of Beijing institute of Microbiology and Epidemiology. It was informed of the objectives, requirements and procedures of the experiments. Before each feeding process, a single dose of a non-steroidal anti-inflammatory agent (NSAID) Aspirin was orally administrated to mice to alleviate the suffering of the mice, following the guidance of IACUC of Beijing institute of Microbiology and Epidemiology.

## Results

### *R. moncacensis* prevalence in ticks and its cultivation from *I. sinensis* hemolymph

**Figure 2:**
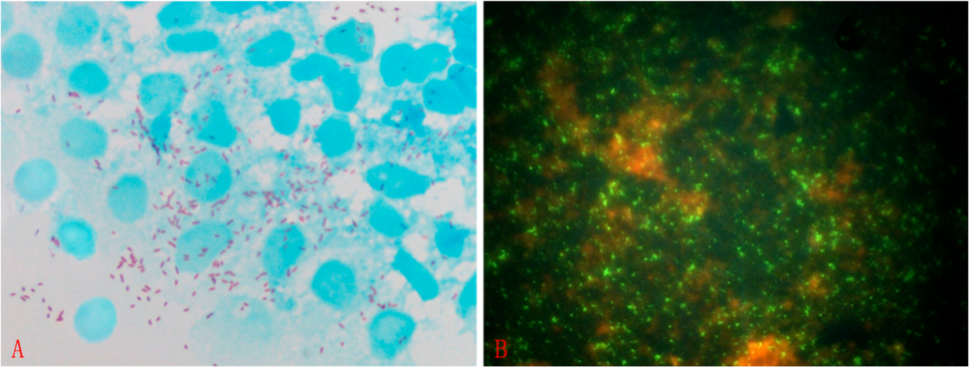
***Rickettsia monacensis*** **cultured from** ***I. sinensis.*** *Rickettsia monacensis* cultured in HEL fibroblast from the hemolymph of *I. sinensis* Guangshan, Henan. (Panel **A**. cell smear stained with Gimenez and Panel **B**. Indirectly Immunofluorescence Assay with commercial antibody of *R. monacensis* produced by Vircell, Spain)*.*

**Figure 3:**
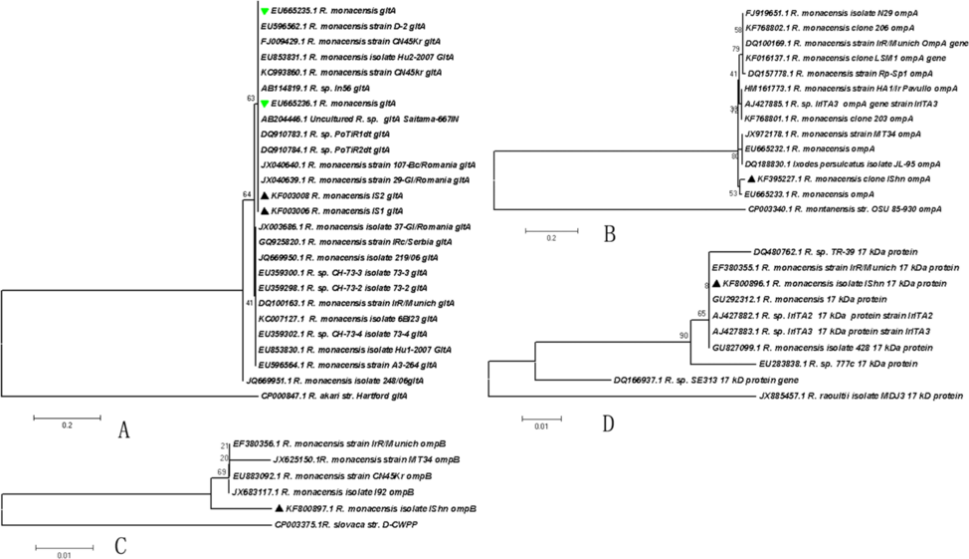
**Phylogenetic tree of** ***R.monacensis.*** Phylogenetic tree of *R.monacensis* based on (panel **A**): 381-bp the citrate synthase (*glt*A), (panel **B**) 533-bp the outer membrane protein A (*omp*A) gene, (panel **C**), 515-bp the outer membrane protein B (*omp*B) gene and (panel **D**) 438-bp 17 kDa protein gene. The tree was calculated by neighbor-joining method using MEGA 5.2 software. Values of the bootstrap support of the particular branching calculated for 10,000 replicates are indicated at the nodes. The variant sequences obtained in this study were designated by accession number and species and/or strain name.

A total of 1503 ticks were classified into 5 species belonging to 4 genera. These included 586 *I. sinensis* (198 ♂♂, 388 ♀♀), 78 *Hae. longicornis* (19 ♂♂, 59 ♀♀), 276 *Hae. flava* (78 ♂♂, 198 ♀♀), 285 *Dermacentor steinii* (167 ♂♂, 118 ♀♀), and 278 *Rhicephalus microplus* (80 ♂♂, 198 ♀♀) (Figure 1). Samples positive for corresponding fragments of both *glt*A and *omp*A of SFG *Rickettsia* spp*.* were considered SFG rickettsia infections. Using this criterion, the expected DNA fragments of *R. monacensis* were detected in 9 of 586 *I. sinensis* ticks, 2 ♂♂ (1.06%) and 7 ♀♀ (1.80%). The difference between sexes was not significant (χ^2^ = 0.894, P > 0.05). No *Hae. longicornis*, *Hae. flava*, *Rh. microplus* or *D. steinii* was positive for *Rickettsia* spp. Similar to the PCR results, cultivation of tick hemolymph to detect *R. monacensis *was only positive for *I. sinensis,* as shown in HEL fibroblasts stained with Gimenez and indirect immunoflourescence assay results (Figure 2). To confirm the positive results of the culture, we sampled cells from the cover slips and used primers targeting *glt*A gene, *omp*A gene, *omp*B gene and 17-kD protein gene respectively and got positive results as expected. All sequences obtained (KF000308 for *glt*A, KF395227 for *omp*A, KF800897 for *omp*B and KF800896 for 17-kDa protein gene) were aligned with known sequences using the BLAST (http://blast.ncbi.nlm.nih.gov/Blast.cgi) program. The best matches (highest identities) occurred with the corresponding sequence coding genes of *R. monacensis*. Nucleotide sequence identities ranged from 99.93% to 99.92% for the *glt*A gene, from 99.09% to 100% for the *omp*A gene, from 99.46 to 100% for the *omp*B gene, and from 99.72% to 100% for the 17-kDa protein gene, indicating that both the homology levels of the sequencesare within species thresholds for *R. monacensis* proposed by Fournier and others [25]. Sequences related to corresponding reference sequences of the universally recognized SFG group *Rickettsia* spp. in Genbank were utilized to construct phylogeny trees, the new sequences were clustered into a separate *R. monacensis* branch (Figure 3).

### Transmission of *R. monacensis*by*I. sinensis*

The potential pathogenicity of *R. monacensis* to *I. sinensis* was evaluated by comparison with a control group, fed with the same volume of PBS solutions through capillary tubes. Over 30 d post CTF, ticks died at approximately the same rate with no significant differences between groups. At 5 d after tick detachment from mice, all hemolymph samples collected from 20 engorged females of *I. sinensis* and blood samples from the infested mice were infected with *R. monacensis*, indicating successful infection by CTF. The replete females were maintained individually until egg production at approximately 21 d. All 15 egg pools from 5 females yielded positive results as did the 15 larval ones, suggesting a 100% TOT infection rate.

We tested for *R. monacensis* infection in *I. sinensis* larvae by TS to nymphs by immersing 300 *Rickettsia* free *I. sinensis* larvae in medium containing *R. monacensis*. At 7 d post immersion, a total of 284 larvae survived and there was no significant difference with the control group treated with a PBS solution. After infesting the C_3_H mice, 186 engorged larvae were harvested. All the 30 sampled engorged larvae were infected with *R. monacensis.* Four weeks later, 145 nymphs were recovered and 29 of 50 sampled nymphs contained *Rickettsia*. This is a 58.0% TS infection rate from larval to nymphal stage (Table 1). We fed 160 *Rickettsia* free *I. sinensis* nymphs with the medium containing *R. monacensis* by CTF to study transmission from nymphs to adults. At 7 d post CTF, 148 nymphs survived and these were not significantly different from the control groups. After feeding on the C_3_H mice, 130 engorged nymphs were harvested. A sample of 30 engorged nymphs was all infected with *R. monacensis.* At 42 d, 88 adults were recovered and 10 of 25 males and 18of 25 females sampled were positive, indicating a 56.0% TS infection rate from nymph to adult (Table 1). All 17 mice fed by infected *I. sinensis*, 5 by larvae, 6 by nymphs and 6 by adults, were *Rickettsia* positive, as determined by PCR, indicating the 100% transmitting efficiency from tick to host reservoir (Table 1).

**Table 1:** **Results for transmission experiments**

**Stage**	**Group**	**No. survived/treated**	**No. engorged**	**No. infected/sampled**			
				**Engorged**	**Subsequent stage or sex**		**Mice infested**
**From adult to larva**	Infected group^A^	21/30	20	12/12	Egg 15/15*	Larvae 15/15*	5/5
	Control^A^	21/30	20	0/11	Egg0/15*	Larvae0/15*	0/5
**From larva to nymph**	Infected group^B^	300/300	284	30/30	Nymph 29/50		6/6
	Control^B^	300/300	282	0/30	Nymph 0/50		0/6
**From nymph to adult**	Infected group^C^	148/160	130	28/30	Male 10/25	Female 18/25	6/6
	Control^C^	145/160	129	0/30	Male 0/25	Female0/25	0/6

Note: ^A^female; ^B^larva; ^C^nymph; *100 individuals as a pool.

## Discussion

Mediterranean Spotted like Fever and its pathogen *R. monacensis* has been well characterized in previous studies and natural infection of *R. monacensis* in many tick species has also been confirmed in many countries. However, the details of the maintenance and transmission of *R. monacensis* in ticks remain incomplete. In Eurasia, *I. ricinus* has been regarded as the vector in many MSF like cases. Due to its close morphological and phylogenic relationships with *I. ricinus*, in America *I. scapularis,* was considered a vector candidate in assessing the possible transmission mechanism by Baldridge *et al.* [26]. Using green fluorescent protein (GFP) expressing *R. monacensis* Rmona658, they demonstrated the transmission of *R. monacensis* in *I. scapularis* from larvae to nymphs and nymphs to adults. However, TOT and horizontal transmission failed. Rmona658 did not establish in small mammal hosts by the feeding of either *I. scapularis* nymphs or adults. Thus no other tick species except *I. ricinus* has been demonstrated to be a vector of *R. monacensis* until the present report.

Our results estimate the natural infection of *I. sinensis* with *R. monacensis* in Chinese locations, suggesting that MSF like rickettsiosis occurs in both central and southern China. The potential threat of *R. monacensis* should be considered in differential diagnosis in spotted fever patients. Considering natural infection and experimental transmission evidence from TS, TOT and horizontal transmission protocols, *I. sinensis* appears to be a competent vector. *I. sinensis* has frequently been recorded feeding on residents and tourists in central and southern China [27,28] so the potential health risks of *I. sinensis* and *R. monacensis* should be recognized by public health authorities. The vector competence of *I. sinensis* helps to explain the trend of southward expanding range of MSF-like rickettsiosis in China. The valid geographic range of *I. sinensis* is not limited to the areas sampled in this report [27,29], therefore, MSF-like rickettsiosis might occupy larger geographical areas in China.

In our survey, *R. monacensis* was only found in *I. sinensis;* but it has previously been recorded in *I. persulcatus* [14]*.* No *I. persulcatus* were collected in the present study and this species mainly occurs in the north and northeast China [27]. The southern range limit of *I. persulcatus* is in the Qiling-Taihang-Yanshan Mountains [27,28]. Our survey sites did not include this range and we collected neither *R. sibrica* nor *R. heilongjiangensis*, which often co-occurs with *I. persulcatus.* Thus, we assume misidentification of ticks might have occurred in the report of Li *et al.* [14] because of the morphological similarity of the two species (they belong to the same complex) and despite the fact that temporal and spatial factors and even the low prevalence might contribute to the differences.

*I. sinensis* belongs to the *I. ricinus* species complex which is important in the animal to human transmissionof tick borne pathogens such as, *Borrelia burgdorferi*, *Babesia* protozoans, *Anaplasma phagocytophilum. I. sinensis*is the principal vector of the Lyme disease agent *Borrelia burgdorferi* and related *Borrelia* species in southern China [27]. Because the transmission cycle of *R. monacensis* by *I. sinensis* appears similar to that of *Borrelia* spp, co- infection of humans by *R. monacensis* and other tick borne pathogens might occur in some regions. The co-infection prevalence in human populations and the related public health risks will require further investigation.

## Conclusions

As demonstrated by natural infection and transmission studies, *I. sinensis* is a competence vector for *R. monacensis*, the agent for Mediterranean Spotted Fever like rickettsiosis.
